# Formin-mediated bridging of cell wall, plasma membrane, and cytoskeleton in symbiotic infections of *Medicago truncatula*

**DOI:** 10.1016/j.cub.2021.04.002

**Published:** 2021-06-21

**Authors:** Pengbo Liang, Clara Schmitz, Beatrice Lace, Franck Anicet Ditengou, Chao Su, Eija Schulze, Julian Knerr, Robert Grosse, Jean Keller, Cyril Libourel, Pierre-Marc Delaux, Thomas Ott

**Affiliations:** 1 University of Freiburg, Faculty of Biology, Cell Biology, Schänzlestr. 1, 79104 Freiburg, Germany; 2 University of Freiburg, Medical Faculty, Institute of Pharmacology, Albertstr. 25, 79104 Freiburg, Germany; 3 CIBSS – Centre of Integrative Biological Signalling Studies, University of Freiburg, Schänzlestr. 8, 79104 Freiburg, Germany; 4 Laboratoire de Recherche en Sciences Végétales (LRSV), Université de Toulouse, CNRS, UPS, 24, chemin de Borde-Rouge, 31326 Castanet-Tolosan, France

**Keywords:** root, nodule, symbiosis, actin, formin, infection, rhizobium

## Abstract

Legumes have maintained the ability to associate with rhizobia to sustain the nitrogen-fixing root nodule symbiosis (RNS). In *Medicago truncatula*, the Nod factor (NF)-dependent intracellular root colonization by *Sinorhizobium meliloti* initiates from young, growing root hairs. They form rhizobial traps by physically curling around the symbiont.^1^^,^^2^ Although alterations in root hair morphology like branching and swelling have been observed in other plants in response to drug treatments^3^ or genetic perturbations,^4^, ^5^, ^6^ full root hair curling represents a rather specific invention in legumes. The entrapment of the symbiont completes with its full enclosure in a structure called the “infection chamber” (IC),^1^^,^^2^^,^^7^^,^^8^ from which a tube-like membrane channel, the “infection thread” (IT), initiates.^1^^,^^2^^,^^9^ All steps of rhizobium-induced root hair alterations are aided by a tip-localized cytosolic calcium gradient,^10^^,^^11^ global actin re-arrangements, and dense subapical fine actin bundles that are required for the delivery of Golgi-derived vesicles to the root hair tip.^7^^,^^12^, ^13^, ^14^ Altered actin dynamics during early responses to NFs or rhizobia have mostly been shown in mutants that are affected in the actin-related SCAR/WAVE complex.^15^, ^16^, ^17^, ^18^ Here, we identified a polarly localized *SYMBIOTIC FORMIN 1* (*SYFO1*) to be required for NF-dependent alterations in membrane organization and symbiotic root hair responses. We demonstrate that SYFO1 mediates a continuum between the plasma membrane and the cell wall that is required for the onset of rhizobial infections.

## Highlights

• The SYMBIOTIC FORMIN 1 (SYFO1) specifically regulates symbiotic root hair curling
• SYFO1 directly binds actin and polarizes in responding root hairs
• SYFO1 induces membrane protrusions in cell-wall-devoid protoplasts
• Cell wall association of SYFO1 is indispensable for its function in root hairs

## Graphical abstract

**Figure:**
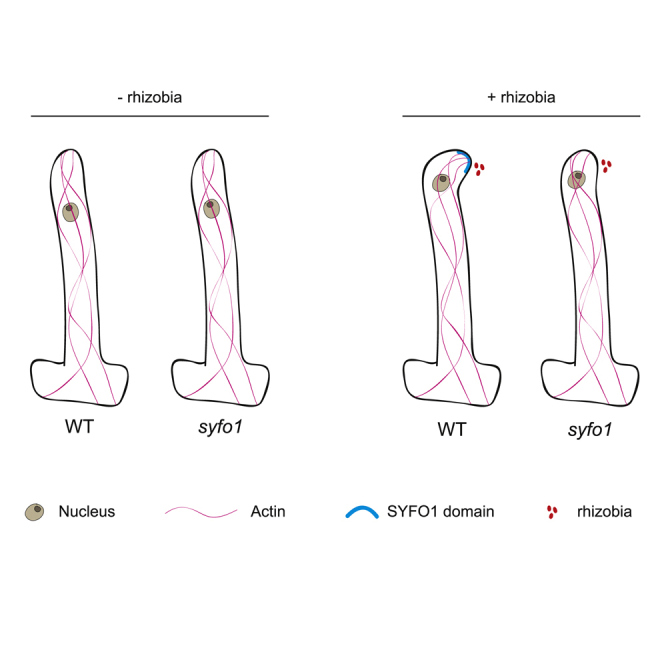

## Teaser

Here, Liang et al. report that the formin SYFO1 specifically regulates polar actin alignments during symbiotic root hair responses in *Medicago truncatula*. Although SYFO1 is able to induce membrane protrusions in protoplasts, its endogenous function requires an association with the cell wall.

## Results and discussion

### Evolutionary and transcriptional patterns identify symbiotically regulated formins

**Figure 1:**
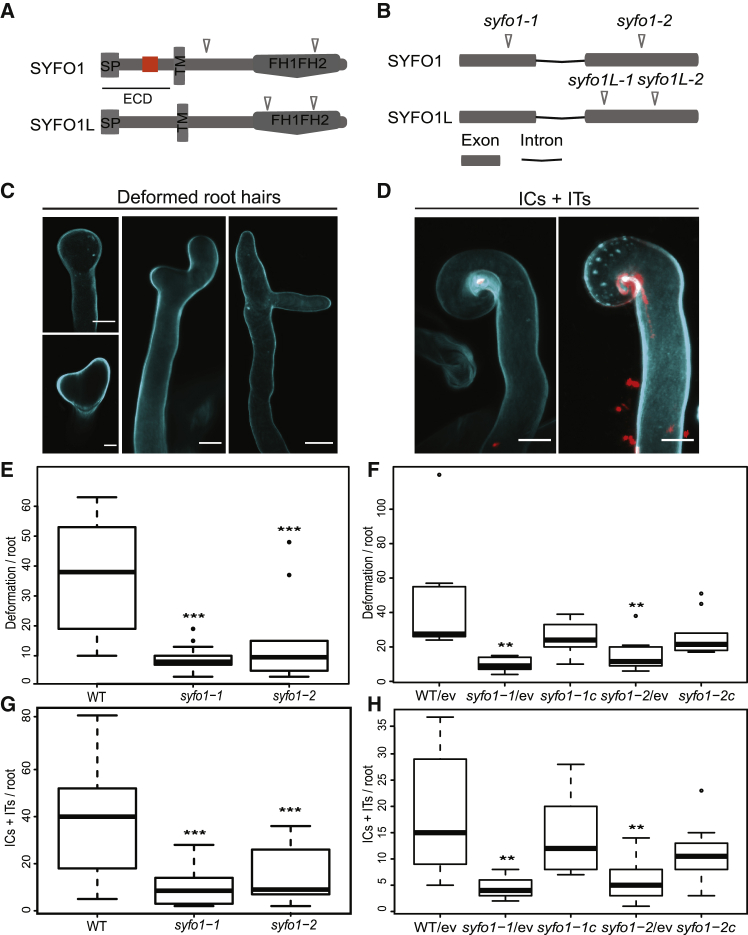
*syfo1* mutants are impaired in symbiotic root hair responses (A and B) Isolation of independent mutant alleles with *Tnt1* transposon insertions mapping to different regions of the *SYFO1* and *SYFO1L* protein (A) and the gene (B) models. ECD, extracellular domain; SP, signal peptide; TM, transmembrane domain; orange box, proline-rich repeat (PRR). (C and D) Images show cell wall stained by Calcofluor white in deformed root hairs (C), infection chambers (ICs), and infection threads (ITs) (D) on wild-type plants to illustrate the scored structures. Scale bars indicate 10 μm. (E and F) *syfo1* mutants show significantly reduced responsiveness to the presence of compatible rhizobia when assessing root hair deformations in mutants (E) and genetically complemented roots by introducing a genomic version of the full-length *SYFO1* gene driven by the endogenous *SYFO1* promoter (*syfo1-1c* and *syfo1-2c*) with an empty vector (ev) transformation control aside (F). (G and H) Infection-related structures, such as ICs + ITs, were scored in mutants (G) and complemented roots (H). Asterisks indicate a significant statistical difference based on a Tukey-Kramer multiple-comparison test with ^∗∗^p < 0.01 and ^∗∗∗^p < 0.001. Data are shown as mean ± SE with independent 9–14 plants for phenotypical analysis and 10 plants for complementation analysis. Phenotypes were scored at 5 dpi. See also Figures S1–S3 and Table S1.

Based on the presence of a conserved formin homology 2 (FH2) domain, we identified 20 candidates in the *Medicago truncatula* genome (Table S1). Using publicly available transcriptome data, two of them (*Medtr5g036540.1* and *Medtr8g062830.1*) were found to be transcriptionally upregulated during early stages of symbiotic interactions.^19^ We independently verified these data using qRT-PCR with *Medtr5g036540.1* being induced by about 60-fold at 1 day post inoculation (dpi) of roots with *S. meliloti* while only a weak induction could be confirmed for *Medtr8g062830.1* at 5 dpi (Figures S1A and S1E). Therefore, we named the genes *SYMBIOTIC FORMIN 1* (*SYFO1*) (*Medtr5g036540.1*, *MtrunA17_Chr5g0414941* in the v5r1.6 *M. truncatula* genome version) and SYFO1-like (*SYFO1L*) (*Medtr8g062830.1, MtrunA17_Chr8g0364331*). Both encoded proteins contain a predicted signal peptide in the extracellular domain followed by a single-span transmembrane domain and a FH1FH2 domain in the cytoplasmic side (Figure 1A).

To spatially resolve the observed transcriptional patterns for *SYFO1*, we generated a fluorescent reporter where a nuclear localized tandem GFP was driven by the endogenous *SYFO1* promoter (*ProSYFO1-NLS-2xGFP*). Consistent with the qRT-PCR results (Figures S1A and S1E), the *SYFO1* promoter was activated at 1 dpi in root hairs and cortical cells (Figures S1B and S1C), while no activity was observed in the absence of the symbiont.

The *SYFO1/1L* clade contains genes from all eudicot species included in the analysis, with *SYFO1* and *SYFO1L* deriving from the Papilionoideae duplication and the three *A. thaliana* co-orthologs (*AtFH4*, *AtFH7*, and *AtFH8*) likely derived from the Brassicaceae triplication (Figure S1D). AtFH4 and AtFH8 resemble a similar protein domain structure compared to SYFO1 and SYFO1L, whereas AtFH7 has lost the signal peptide and displays an altered transmembrane domain. We also found *SYFO1* (in common bean, *Phvul.002G077100.2*) or *SYFO1L* (in *Lotus japonicus*, *Lj0g3v0115049.1*) upregulated during nodulation, pinpointing a potential shared function in nodulation within this clade.

Based on the phylogeny, we tested for relaxed (red dots) or intensified (blue dots) selective pressure acting on different branches of interest in eudicot sequences (Figure S1D; Table S3). We identified a relaxed selective pressure indicated by a switch on ratio of non-synonymous (dN) versus synonymous (dS) mutations (dN/dS) on the Fabales clade compared to the background dN/dS (K = 0.54, LRT = 22.32, and p < 2.31e−06; Table S3). In contrast, the Brassicaceae clade is under strong selective pressure intensification (K = 9.34, LRT = 202.89, and p < 1e−16; Table S3) that is not even relaxed by the triplication of the branch in this family. The relaxed selection pressure detected on the Fabales may reflect neofunctionalization that would have occurred for the recruitment of SYFO1/1L in root nodule symbiosis, possibly together with the symbiont switch from *Frankia* to rhizobia. Interestingly, the corresponding orthologs of *Parasponia andersonii*, a non-legume tree that forms a Frankia-type symbiosis with rhizobia, are not induced during the symbiotic interaction. In addition, following the duplication of SYFO1 and SYFO1L in Papilionoideae, an intensification of selection was observed on the SYFO1 clade (K = 6.46, LRT = 52.89, and p = 3.53e−13; Table S3), although the SYFO1L clade experienced relaxed selection (K = 0.45, LRT = 40.61, and p = 1.86e−10; Table S3). This further supports a possible functional specialization of the SYFO1 formin in legumes.^20^ The lack of a SYFO1 ortholog in *Lotus japonicus*, however, might be either due to genome assembly issues or to the actual loss of the gene in Lotus but would need further investigation.

### SYFO1 controls rhizobial infection and root hair responses

To genetically assess the function of SYFO1 and SYFO1L, we identified two independent *Tnt1* transposon insertion lines for *SYFO1* (*syfo1-1* [NF9730] at 485 bp and *syfo1-2* [NF9495] at 1,834 bp downstream of the start codon) and *SYFO1L* (*syfo1L-1* [NF20350] at 1,279 bp and *syfo1L-2* [NF15608] at 1,370 bp downstream of the start codon) in *Medicago truncatula* R108 (Figures 1A and 1B). Endogenous transcripts of *SYFO1* and *SYFO1L* were significantly reduced in both lines when scored in roots 1 dpi and in uninoculated roots, respectively (Figures S1F and S1G). However, phenotypically, only the *syfo1-1* and *syfo1-2* alleles showed a significant nodulation phenotype developing fewer nodules per root at 3 weeks post-inoculation (wpi), with about half of them being elongated or spherical but remaining white (both being scored as “aborted”), indicative of non-functional nodules (Figures S2A–S2E). In contrast, patterns of infected nodule cells in *M. truncatula* wild-type (WT) and *syfo1L-1* and *syfo1L-2* mutant nodules were undistinguishable (Figures S2F and S2G). The infection zones were found to be reduced in white but elongated (Figure S2H) or spherical (Figure S2I) *syfo1* mutant nodules. The latter failed to maintain a persistent meristem (Figure S2I). Therefore, we selected SYFO1 as the prime candidate of interest.

**Figure 2:**
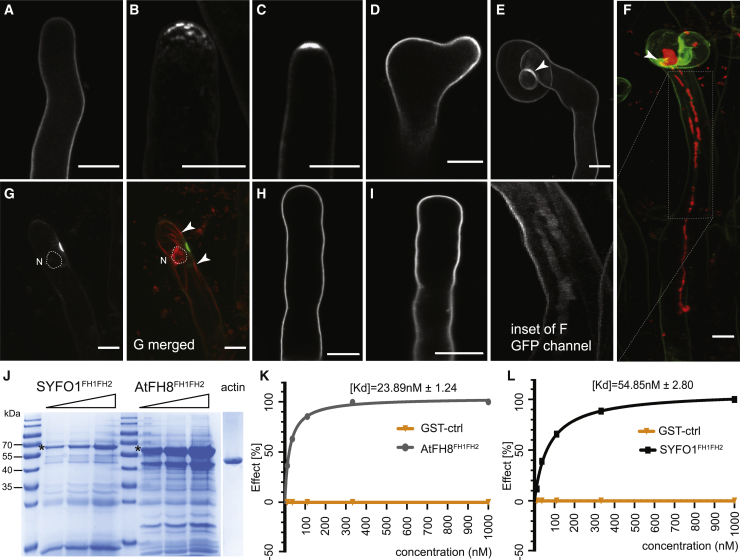
SYFO1 functions as a symbiotic polarity factor in root hairs (A) SYFO1-GFP localizes homogeneously to the PM of root hairs under mock conditions. (B–E) At 2 dpi with *S. meliloti*, SYFO1 transiently polarizes at subapical (B) and apical regions (C) of root hairs before distributing equally along the PM during root hair deformation (D) and curling (E). The arrowhead marks cell wall autofluorescence around the IC (E and F). (F) SYFO1 remains on the IT membrane (see also inset). (G) Co-localization between SYFO1-GFP and the actin marker ABD2:mCherry (see also G merged). The nucleus encircled with a dashed line in (G) and (G merged) is based on corresponding transmitted light image (not shown). The arrowheads point toward actin bundles orienting toward a nucleation center at the apical shank of the root hair. (H) No polar enrichment was observed upon inoculation on the SYFO1^ECD/TMD^-GFP transformed root hairs. (I) Expression of *SYFO1*^*ΔPRR*^*-GFP* resulted in a stable fusion protein that homogeneously distributed along the cell periphery. (J) Purified SYFO1^FH1FH2^ and AtFH8^FH1FH2^ were analyzed by Coomassie blue staining of SDS-PAGE gels. Loading of increasing volumes for each is indicated by triangle form; asterisk (^∗^) indicates the respected products. As control, 5 μg of rabbit muscle actin was loaded. (K) Dose response curves for AtFH8^FH1FH2^ (gray) or a glutathione S-transferase (GST)-control (orange) to a CM5-Chip coupled to rabbit muscle actin. (L) Dose-response curves for SYFO1^FH1FH2^ (black) or GST-control (orange) to a CM5-Chip coupled to rabbit muscle actin. For each condition, five different concentrations were tested (1 μM, 333 nM, 111 nM, 37 nM, and 12.3 nM) in 3 independent biological replicates (n = 3) for SYFO1^FH1FH2^ and 2 independent biological replicates for AtFH8^FH1FH2^ (n = 2). Data are mean ± SD. Scale bars indicate 10 μm (A–F). See also Figure S3.

Although several cytoskeleton-related mutants, but not *scarn*,^17^ exhibit impaired root hair growth, this was not observed for *syfo1-1* and *syfo1-2* (Figures S3A–S3C). Neither did we observe any differences in actin arrangement in growing root hairs within the symbiotically susceptible infection zone^2^ under non-inoculated conditions (Figures S3D–S3J). These data demonstrate that SYFO1 is not required for normal root hair development under non-symbiotic conditions, even though SYFO1 localizes to root hairs even under non-inoculated conditions (Figure 2A). As *SYFO1* transcripts were upregulated at 1–3 dpi (Figure S1A), a stage where we observed most root hairs responding to the presence of the symbiont by root hair deformation (1 to 2 dpi; Figure 1C) and curling (2 to 3 dpi; Figure 1D), we phenotypically assessed *syfo1* mutants at these stages. Both mutant alleles showed significantly fewer deformations (Figure 1E) as well as total infection chambers and infection threads (Figure 1G) at the respective time points. Further dissection of the phenotype indicated that the individual numbers of infection chambers (ICs) (Figure S3K) and infection threads (ITs) (Figure S3L) were significantly decreased in the mutants. However, the size of both ICs and ITs remained unaltered in the mutants compared to the WT (Figures S3M and S3N). Both phenotypes were rescued in independent transgenic roots expressing a *ProSYFO1:SYFO1-GFP* (e.g., *syfo1-1c*) construct in these mutant backgrounds (Figures 1F and 1H), demonstrating that the *syfo1* mutations caused the observed phenotypes. The fact that the number of ICs was higher in Lotus *scarn* mutants compared to WT plants^17^ places SCARN downstream of SYFO1. Because the bacterial colonization phenotypes in nodules are comparable between both mutants, both may also contribute to actin function at this stage.

Interestingly, cell-type-specific expression of *SYFO1* using a root-epidermal-specific expansin promoter (ProEXP) of *M. truncatula*^21^ failed to rescue the deformation phenotype (Figure S3O), indicating that the transcriptional landscape mediated by the *SYFO1* promoter is crucial for SYFO1 function. Beside the transcriptional pattern, Rhizobium-triggered root hair responses specifically require the SYFO1 protein, as expression of the closely related SYFO1L and the *Arabidopsis* AtFH8 formin under control of the *SYFO1* promoter did not rescue the *syfo1* mutant phenotype (Figure S3P).

As it was previously demonstrated that the inoculation of *L. japonicus* root hairs with its symbiont *Mesorhizobium loti* resulted in a polarization and bundling of actin filaments with a strong accumulation of F-actin in the root hair tip,^16^^,^^17^ we tested whether this pattern is affected in *syfo1* mutants. In the absence of *S. meliloti*, longitudinal actin filaments were observed in young growing root hairs within the infection zone of *M. truncatula* WT plants (Figure S3R). In agreement with the observations in *L. japonicus*, actin strongly bundled and polarized with an accumulation of F-actin at the apex (Figures S3S and S3T) or occasionally at the apical shank of responding root hairs (Figure S3U) in 88% of all root systems of WT plants inoculated with *S. meliloti* (Figure S3V). However, this pattern was strongly reduced in both *syfo1* mutant alleles where only about 30% of all tested roots contained root hairs responding with the above-mentioned pattern (Figure S3V).

### SYFO1 associates with polar actin assemblies under symbiotic conditions

Because our data indicated a symbiosis-specific role of SYFO1 in root hair polarization, we investigated spatial and temporal dynamics of SYFO1 at subcellular resolution. As mentioned above, we observed a weak homogeneous signal in roots expressing a *ProSYFO1:SYFO1-GFP* construct at the PM of root hairs in the absence of rhizobia (Figure 2A). The underlying low, basal expression was also detected by qRT-PCR (Figure S1E), although it was most likely too weak when using the nuclear-localized GFP reporter to test promoter activity (Figure S1B). Interestingly, SYFO1 strongly accumulated in subapical and apical foci at root hair tips prior to deformation at 2 dpi with *S. meliloti* (Figures 2B and 2C), which strongly resembled actin patterns observed upon Nod factor (NF) application, as reported earlier.^16^ In root hairs that morphologically responded by deformation (Figure 2D) and curling (Figure 2E), SYFO1 distributed again along the PM with only mild accumulations at the apical region (Figure 2D). Very weak SYFO1 signal was also detected along the infection thread membrane (Figures 2F and 2F, inset). The signal around the infection chamber (Figures 2E and 2F, indicated by arrowheads) is due to cell wall autofluorescence as described earlier.^1^^,^^2^^,^^7^^,^^8^ This makes it unlikely that SYFO1 plays primary roles during IT growth.

When co-localizing actin and SYFO1 (here: *ProUbi-SYFO1-GFP*), we frequently observed transient SYFO1 accumulations in close proximity to enlarged nuclei, a hallmark for symbiotically activated root hairs,^22^ with actin bundles orienting toward a nucleation center at the apical shank of the root hair (Figure 2G and G merged). In order to test whether the FH1FH2 domain of SYFO1 directly associates with G-actin, we recombinantly expressed and isolated this domain from *E. coli* (Figure 2J). We additionally used the *Arabidopsis* formin AtFH8^FH1FH2^ as a positive control.^5^ Applying surface plasmon resonance, we were able to detect a direct interaction between G-actin and SYFO1^FH1FH2^ with a K_d_ of 54.85 nM compared to a K_d_ of 23.89 nM found for AtFH8^FH1FH2^ (Figures 2K and 2L), suggesting a high-affinity binding of SYFO1^FH1FH2^ to actin.

### Ligand-dependent morphological change mediated by SYFO1

**Figure 3:**
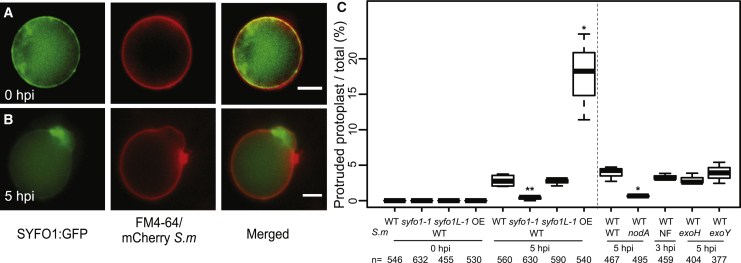
SYFO1 is required for NF-induced membrane protrusions in *M. truncatula* protoplasts (A) Protoplasts of a *M. truncatula* ROC constitutively expressing SYFO1 and counterstained with the styryl dye FM4-64 show localization of SYFO1 to the PM with some additional cytoplasmic signal at 0 hpi. (B) Focal membrane protrusions with centrally accumulated SYFO1 were found at 5 hpi with mCherry-expressing *S. meliloti* (*S.m.*). (C) Quantification of membrane protrusions in protoplasts using different genetic backgrounds: protoplasts isolated from seedling roots of *syfo1-1/syfoL1-1* mutants or protoplasts isolated from ROC overexpressing (OE) SYFO1-GFP. Protoplasts were inoculated with mCherry-expressing *S.m.* (WT) or different *S.m.* mutants. Asterisks indicate a significant statistical difference based on an ANOVA followed by a Fisher least significant difference (LSD) test, with ^∗^p < 0.05 and ^∗∗^p < 0.01. Data are shown as mean ± SE of 3 independent biological replicates with n indicating the total number of protoplasts being scored. NF, Nod factor. Scale bars indicate 5 μm (A and B). See also Figure S4.

Using the polarity marker BREAKING OF ASYMMETRY IN THE STOMATAL LINEAGE (BASL) in BY-2 protoplasts, it was recently shown that these isolated, spherical cells have an intrinsic polarity.^23^ This fact additionally requires a polarization of the secretion machinery for delivering membrane material and proteins. To test the effect of polarized SYFO1 secretion in protoplasts, we generated a transgenic *M. truncatula* root organ culture (ROC) constitutively expressing SYFO1-GFP. Here, SYFO1 resided in the PM, where it co-localized with the membrane stain FM4-64 with some larger polar accumulations in the periphery (Figure 3A). Interestingly, inoculation of these protoplasts with *S. meliloti* for 5 h reproducibly resulted in focal membrane outgrowths with central SYFO1 accumulations (Figure 3B). To unambiguously verify that SYFO1 is the key driver of these protrusions, we isolated protoplasts from our *syfo1-1* and *syfo1L-1* mutants. No protrusions were found in the absence of rhizobia in any of the used genotypes. Upon inoculation of protoplasts with *S. meliloti*, those expressing WT *SYFO1* (WT, *syfo1L-1*) or overexpressing *SYFO1-GFP* (OE) developed protrusions, while they were entirely absent on protoplasts isolated from the *syfo1-1* mutant (Figure 3C). This demonstrates that SYFO1 is required for Rhizobium-induced focal membrane deformations. To further exploit the molecular triggers of this morphological change, we tested different rhizobial strains being defective for NF (*nodA*) or exopolysaccharide (EPS) (*exoH* and *exoY*) production.^24^^,^^25^ Interestingly, only the *nodA* mutant failed to induce membrane protrusions on WT protoplasts, although application of isolated NFs for 3 h as well as *exoH* and *exoY* strains for 5 h resulted in the same number of membrane outgrowths compared to those induced by *S. meliloti* 2011 (Figure 3C). Taken together, these data indicate that the formation of SYFO1-dependent membrane protrusions are triggered by NFs. We currently hypothesize that these protrusions rely on a targeted secretion of endoplasmic reticulum (ER) and Golgi vesicles to intrinsic polar domains. As rhizobia stimulate active exocytosis of membrane lipids and proteins that are required for initial root hair responses and later for building and maintaining infection threads in legumes, we assume that SYFO1 is actively secreted to these sites and potentially increases local membrane areas. Similar secretory patterns toward sites of microbial attachment and possibly penetration have been described for the *Arabidopsis* FORMIN4.^26^

### A SYFO1-mediated cell wall-plasma membrane-cytoskeleton continuum is required for symbiotic responses in root hairs

**Figure 4:**
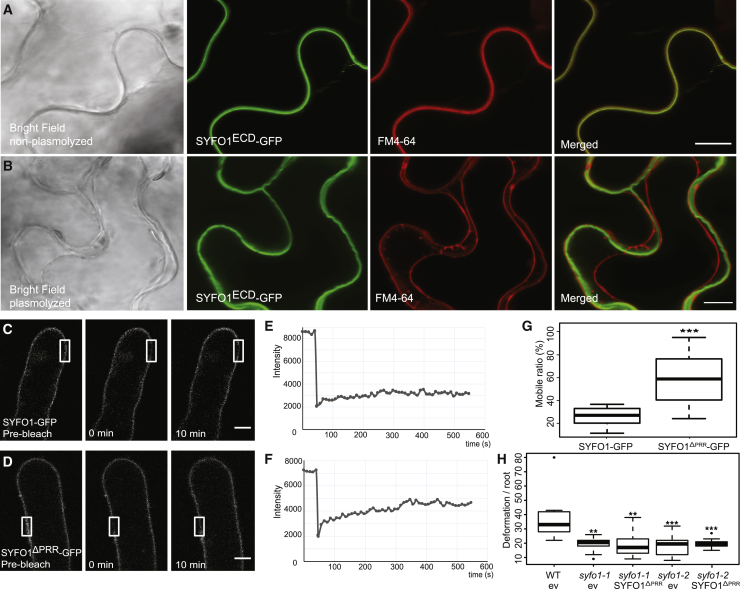
Cell wall association of SYFO1 is essential for its function (A and B) The constitutively expressed ECD of SYFO1 labeled the cell periphery in non-plasmolyzed cells (A) and remained at the cell wall upon plasmolysis (B). Arrowheads and arrows mark the cell wall and the retracted plasma membrane, respectively. (C–F) FRAP experiments on roots hairs revealed a low mobility of full-length SYFO1 (C and E), although deletion of the PRR resulted in an increased mobility of the protein (D and F). (G) Quantification of the mobile fractions of SYFO1 (n = 17 regions of interest [ROIs] from 4 independent plants) and SYFO1^ΔPRR^ (n = 12 ROIs from 4 independent plants); asterisks indicate a significant statistical difference based on a Student’s t test. (H) The SYFO1^ΔPRR^ variant failed to genetically complement both *syfo1* mutant alleles in comparison to roots transformed with the empty vector (ev) scoring n = 10 independent root systems per genotype. Asterisks indicate a significant statistical difference based on a Tukey-Kramer multiple-comparison test with ^∗∗∗^p < 0.001 and ^∗∗^p < 0.01. Data are shown as mean ± SE. Scale bars indicate 10 μm (A–D). See also Figure S4.

As actin binding of formins is generally mediated by the FH1FH2 domain that is also present in the cytosolic region of SYFO1 (Figure 1A), we examined the extracellular domain (ECD), which is less prominently found among formin proteins. Further sequence analysis of the SYFO1^ECD^ revealed the presence of a proline-rich repeat (PRR) (e.g., Ser-Pro-Pro-Pro-Ser-Pro-Ser-Ser [SPPPSPSS]) between the signal peptide and the transmembrane domain, which resembles a canonical motif of extensin proteins that have been proposed to contribute to cell wall architecture and tensile strength.^27^^,^^28^ To investigate a possible role in cell wall association of the ECD, we fused the 82 amino acids of the SYFO1 ECD to GFP (SYFO1^ECD^-GFP) and expressed it in *Nicotiana benthamiana* leaf epidermal cells. Fluorescence was found in the cell periphery in control cells where it co-localized with the styryl dye FM4-64 (Figure 4A). Upon plasmolysis, the SYFO1^ECD^-GFP signal remained predominantly associated with the cell wall, although the FM4-64-labeled PM retracted from the cell wall (Figure 4B). Interestingly, expression of a SYFO1^ECD/TMD^-GFP peptide (this construct included the transmembrane domain) in young root hairs resulted in a uniform labeling of the cell periphery without any clear sign of polarity upon inoculation with *S. meliloti* (Figure 2H). This is in contrast to the full-length SYFO1 protein that transiently accumulated in the tip of growing roots hairs upon inoculation (Figures 2B and 2C). These findings support the hypothesis that full-length SYFO1 contributes to polarity rather than just being secreted in a polarity domain.

In order to test whether the extensin-like motif contributes to the lateral immobilization of SYFO1 via cell wall anchoring, we performed fluorescence recovery after photobleaching (FRAP) experiments on root hairs constitutively expressing a full-length SYFO1 (SYFO1-GFP) or a mutant variant where we deleted the proline-rich repeat (SYFO1^ΔPRR^-GFP). A clear fluorescent signal at the cell periphery indicated stability of this protein variant in *M. truncatula* root hairs (Figure 2I). Our FRAP experiments revealed a slow recovery of the bleached region and a mobile fraction of about 24% for WT SYFO1, whereas the mobile fraction for SYFO1^ΔPRR^ was significantly higher (57%; Figures 4C–4G). This clearly indicates that the PRR segment within the SYFO1 ECD anchors this formin protein to the cell wall, as also described for the *Arabidopsis* formin AtFH1.^29^ To address whether the cell wall association is required for SYFO1 function, we conducted genetic complementation experiments, and we generated transgenic roots expressing SYFO1^ΔPRR^ under the control of the native *SYFO1* promoter in our *syfo1-1* and *syfo1-2* alleles. In contrast to the full-length SYFO1 (Figures 1F and 1H), the deletion of the PRR fully abolished the ability to complement the root hair deformation (Figure 4H), as well as infection chambers and infection threads (Figure S3Q), phenotypes of *syfo1* mutants, demonstrating that an SYFO1-mediated cell wall-plasma membrane-actin continuum is required for symbiotic responsiveness of root hairs in *M. truncatula*.

## STAR★Methods

### Key resources table

**Table:** 

REAGENT or RESOURCE	SOURCE	IDENTIFIER
**Bacterial and virus strains**		

*Sinorhizobium meliloti 2011*	Lerouge et al.^30^	N/A
*Agrobacterium rhizogenes* strain ARqua1	Boisson-Dernier et al.^31^	N/A
*S. meliloti* strain *exoH*	Bertram-Drogatz et al.^24^	N/A
*S. meliloti* strain *exoY*	Bertram-Drogatz et al.^24^	N/A
*S. meliloti* strain *nodA*	Southwick et al.^25^	N/A
*Escherichia coli* strain BL21 CP	Thermo Fisher Scientific, https://www.thermofisher.com	N/A

**Chemicals, peptides, and recombinant proteins**		

Alexa Fluor 488 Phalloidin	Thermo Fisher Scientific, https://www.thermofisher.com	Cat# A12379
FM4-64	Thermo Fisher Scientific, https://www.thermofisher.com	Cat# T13320
Amoxicillin	Sigma-Aldrich, https://www.sigmaaldrich.com	Cat# A8523
Plant Total RNA-Kit	Sigma-Aldrich, https://www.sigmaaldrich.com	Cat#STRN250-1KT
DNase I	Thermo Fisher Scientific, https://www.thermofisher.com	Cat# EN0521
SuperScriptIII reverse Transcriptase (Invitrogen)	Thermo Fisher Scientific, https://www.thermofisher.com	Cat#18080093

**Experimental models: Organisms/strains**		

*Medicago truncatula* cultivar Jemalong	Heritage Seeds Pty, Adelaide, AU	Jemalong
*Medicago truncatula* ecotype R108 Tnt1 insertion line NF9730 (*syfo1-1*), originally obtained from Noble Research Institute LLC, Ardmore, USA.	This paper and Tadege et al.^32^	NF9730
*Medicago truncatula* ecotype R108 Tnt1 insertion line NF9495 (*syfo1-2*), originally obtained from Noble Research Institute LLC, Ardmore, USA.	This paper and Tadege et al.^32^	NF9495
*Medicago truncatula* ecotype R108 Tnt1 insertion line NF20350 (*syfo1L-1*), originally obtained from Noble Research Institute LLC, Ardmore, USA.	This paper and Tadege et al.^32^	NF20350
*Medicago truncatula* ecotype R108 Tnt1 insertion line NF15608 (*syfo1L-2*), originally obtained from Noble Research Institute LLC, Ardmore, USA.	This paper and Tadege et al.^32^	NF15608

**Oligonucleotides**		

For qRT-PCR and genotyping oligos see Table S4	This paper	N/A

**Recombinant DNA**		

Sub-cloning for LIII expression pattern construct: ProSYFO1:NLS-2xGFP	This paper	N/A
Sub-cloning for LIII FRAP construct, protoplast localization: ProUbi:SYFO1:GFP	This paper	N/A
Sub-cloning for LIII plasmolysis construct: ProUbi:SYFO1^ECD^:GFP	This paper	N/A
Sub-cloning for LIII localization construct: ProSYFO1:SYFO1^ECD/TDM^:GFP	This paper	N/A
Sub-cloning for LIII FRAP construct: ProUbi:SYFO1^ΔPRR^:GFP	This paper	N/A
Sub-cloning for LIII complementation construct: ProSYFO1:SYFO1^ΔPRR^:GFP	This paper	N/A
Sub-cloning for LIII localization construct: ProSYFO1:SYFO1:GFP	This paper	N/A
Sub-cloning for LIII actin marker construct: Pro35S:mCherry:ABD2:mCherry	This paper and Sheahan et al.^33^	N/A
Sub-cloning for LIII construct module as prescreen marker: ProUbi:NLS-2xCerulean	Liang et al.^34^	N/A
Sub-cloning for LIII construct module as prescreen marker: ProUbi:NLS-mCherry	Liang et al.^34^	N/A
Expression pattern analysis: ProSYFO1:NLS-2xGFP // ProUbi:NLS-mCherry	This paper	N/A
FRAP full length: ProUbi:SYFO1:GFP// ProUbi:NLS-mCherry	This paper	N/A
Plasmolysis / localization: ProUbi:SYFO1^ECD^:GFP// ProUbi:NLS-mCherry	This paper	N/A
FRAP truncated version: ProUbi:SYFO1^ΔPRR^:GFP// ProUbi:NLS-mCherry	This paper	N/A
Complementation: ProSYFO1:SYFO1^ΔPRR^:GFP// ProUbi:NLS-mCherry	This paper	N/A
Localization and complementation: ProSYFO1:SYFO1:GFP// ProUbi:NLS-mCherry	This paper	N/A
Co-localization between actin and SYFO1: ProUbi:SYFO1:GFP// Pro35S:mCherry:ABD2:mCherry	This paper	N/A
Localization: ProSYFO1:SYFO1^ECD/TMD^:GFP// ProUbi:NLS-mCherry	This paper	N/A
Recombinant protein purification: 6xHis-AtFH8^FH1FH2^	This paper	N/A
Recombinant protein purification: 6xHis-SYFO1^FH1FH2^	This paper	N/A
Vector pDEST17 for recombinant protein purification	Thermo Fisher Scientific, https://www.thermofisher.com	Cat#C600003

**Software and algorithms**		

All statistical tests have been carried out using the IBM SPSS Statistics software	IBM SPSS Statistics, Version 26	https://www.ibm.com/analytics/spss-statistics-software
All boxplots have been generated using Rstudio	Rstudio, Version 1.3.1073	https://www.rstudio.com
Adobe Illustrator was used for editing and typesetting	Adobe Illustrator, Adobe Illustrator CS7	https://www.adobe.com/products/illustrator.html
ZEN (black version) was used for FRAP data processing	ZEN 2.3	https://www.zeiss.com/microscopy/int/products/microscope-software/zen.html
tBLASTn	tBLASTn v2.8.1+	https://ftp.ncbi.nlm.nih.gov/blast/xecutable/blast+/LATEST
SymDB database	SymDB database	http://symbiogenomesdb.uv.es
MAFFT	MAFFT v7.407	https://mafft.cbrc.jp/alignment/server
trimAl	trimAl v1.4 rev22	http://trimal.cgenomics.org
IQ-TREE	IQ-TREE	http://www.iqtree.org/release/v1.4.2/
ModelFinder	ModelFinder	http://iqtree.cibiv.univie.ac.at
signal v5.0	signal v5.0	http://www.cbs.dtu.dk/services/SignalP
TMHMM	TMHMM v2.0c	http://www.cbs.dtu.dk/services/TMHMM/
RELAX program	RELAX program	http://www.datamonkey.org/RELAX
PAL2NAL program	Suyama et al.^35^	http://www.bork.embl.de/pal2nal/

### Resource availability

#### Lead contact

Further information and requests for resources and reagents should be directed to and will be fulfilled by the Lead Contact, Thomas Ott (Thomas.Ott@biologie.uni-freiburg.de).

#### Materials availability

All constructs newly generated in this study are listed in the Key resources table.

#### Data and code availability

The published article includes all datasets generated or analyzed during this study.

### Experimental model and subject details

This study used the model legume *Medicago truncatula*, its corresponding symbiont *Sinorhizobium meliloti* 2011. Transgenic roots were generated by using *Agrobacterium rhizogenes* strain ARqua1^31^ and recombinant proteins was obtained from *Escherichia coli* strain BL21 CP.

If not stated differently, plants were at 24°C in a 16/8 long-day cycle and a light intensity of 85 mol^∗^m^-2∗^s^-1^ either on Fahraeus medium under sterile conditions or in a vermiculite and sand mixture as specified below.

### Method details

#### Plant growths and phenotypical analysis

For phenotypical analysis *Medicago truncatula* wild-type R108, *syfo1-1*, *syfo1-2*, *syfo1L-1* and *syfo1L-2* seeds were scarified for about 15 minutes in sulfuric acid, washed six times in sterile water, sterilized in bleach solution for 1 minute and washed again six times with sterile water before being sown on 1% agar plates for germination and kept in darkness at 4°C for 3 days for stratification. Germination was allowed for up to 24 hours at 24°C and a light intensity of 85 μmol^∗^m^-2∗^s^-1^ before transferring the seedlings to plates containing Fahraeus medium^36^ for 4 days in the presence of 1 mM nitrate before being transferred to a plate culture system without nitrogen for phenotyping studies. Plants were inoculated with 1ml *Sinorhizobium meliloti* 2011 (mCherry) at an OD_600_ of 0.05 (on plates or open pots with 1:1 ratio of vermiculite and sand mixture). Symbiotic responses including root hair deformations, infection chamber formation and IT development were scored 5 dpi of plants with *S. meliloti* on plates. Soil-based nodulation phenotyping samples were harvested and quantified at 3 wpi. Nodules were embedded in 7% low temperature melting agar and sectioned with a thickness of 60 μm using a vibratome.

#### Genotyping of Tnt1 insertion lines and quantitative Real-Time PCR

R0 or R1 seeds of *M. truncatula* R108 *Tnt1* transposon insertion lines^32^ were obtained from the Noble Research Institute (OK, USA) and insertions were verified using primers listed in Table S4. Total RNA of control and insertion lines was extracted from 30-50 mg root material using a commercial kit (Spectrum Plant Total RNA Kit, Sigma life science) following the supplier’s instructions. Prior to cDNA synthesis, 1 μg total RNA was subjected to an additional DNaseI treatment. Synthesis of cDNA was conducted as described earlier^34^ using the SuperScriptIII reverse Transcriptase (Invitrogen). For qRT-PCR the cDNAs were diluted 1:10 and 1 μL was used per reaction in a SybrGreen assay (Applied Biosystems). All data were normalized to Ct values of the housekeeping gene ubiquitin^37^ using primers listed in Table S4 and PCR products were confirmed by Sanger sequencing.

#### Hairy root transformation

*M. truncatula* hairy root transformation was performed as previously described^31^ using the *Agrobacterium rhizogenes* strain ARqua1. Plants were transferred weekly to fresh plates containing Fahraeus medium (pH 6.0) supplemented with 0.5 mM NH_4_NO_3_ and followed by 2 days of growth on nitrogen-free Fahraeus medium containing 0.1 μM AVG prior to inoculation. Images for localization studies and root hair phenotyping analyses were taken on plants inoculated for 2 days and 5 days, respectively.

#### Phylogenetic and selective pressure analysis

SYFO1 (Medtr5g036540.1) and SYFO1L (Medtr8g062830.1) protein sequences were used as queries for a tBLASTn v2.8.1+^38^ search against a database of 101 Angiosperms genomes (Table S2, sequences can be downloaded from the SymDB database^39^: http://www.polebio.lrsv.ups-tlse.fr/symdb) with an e-value threshold of 1e-10. Sequences were then aligned using MAFFT v7.407^40^ with default parameters. The resulting alignment was trimmed using trimAl v1.4 rev22^41^ to remove positions containing more than 20% of gaps. The cleaned alignment was then subjected to a Maximum Likelihood (ML) analysis using IQ-TREE v1.6.7^42^ as described here after. First, the best-fitting evolutionary model was tested using ModelFinder.^43^ Then a ML search was performed using 10,000 replicates of SH-aLRT^44^ for testing branches support. The tree was finally visualized and annotated with iTOL v4.4.^45^

Signal peptide and transmembrane domains were predicted from proteins using signal v5.0^46^ and TMHMM v2.0c^47^ respectively using default parameters.

To look for relaxation (K < 1) or intensification (K > 1) of selection acting on different lineages of interest in Eudicots (Table S3), we used the RELAX program.^48^ This method calculates different synonymous and non-synonymous substitution rates (ω = *d*N/*d*S) using the phylogenetic tree topology for both foreground and background branches. Protein sequences from SYFO1 and SYFO1L orthologs were aligned using MUSCLE v3.8.382. We used the *PAL2NAL* program^35^ to convert the protein alignment into a codon alignment. We marked different clades of interest in the full tree (Figure S1D; Table S3) and on NFN and Fabales subtrees with the corresponding CDS sequences mentioned in Table S3. The number of analyzed CDS sequences and positions are presented in Table S3.

#### Construct design

The constructs used in this study were designed using Golden Gate cloning.^49^ 2.5 kb upstream of the SYFO1 start codon were chosen as putative promoter region. A Golden Gate compatible full-length genomic DNA version (Medtr5g036540.1) was synthesized (GENEWIZ, Germany) by removing the *BpiI* and *BsaI* restriction sites via silent mutations. All cloning primers are listed in Table S4. To select transgenic roots a *ProUbi-NLS-mCherry* or *ProUbi-NLS-2xCerulean* cassette was additionally inserted into the different T-DNAs containing the transgenes of choice as previously described.^34^ Level II and level III constructs were assembled based on the principle described earlier.^35^ For the recombinant protein purification cloning, FH1FH2 coding region fragments of AtFH8 (aa 227-760^5^) and SYFO1 (aa 227-798) were synthesized (GENEWIZ, Germany) and cloned in frame with N-terminal 6 × His in pDEST17 vector. The resulting clones were sequenced to ensure the in-frame fusion. An overview about all designed constructs is provided in the Key resources table.

#### Bacterial expression and protein purification

Proteins were purified in the *Escherichia coli* strain BL21 CP using the protocol for C-terminal constructs (mDia-ct) according to Li and Higgs.^50^ Proteins were dialyzed into the following buffer: 2 mM NaPO_4_ (pH 7,0), 150 mM NaCl, 0.1 mM EGTA, 0.1 mM DTT and stored at 4°C. Protein concentration was determined by Bradford assay and SDS-Page.

#### Surface plasmon resonance measurements

Dynamic interaction of > 99% rabbit muscle actin (Cytoskeleton) with protein ligands were analyzed by using Biacore X100 system (Biacore). Two lanes on a CM5-chip were generated by coupling 10 μg/ml actin covalently to one lane using 400 mM N-ethyl-N-dimethylaminpropyl-carbodiimide (EDC) and 100 mM N-hydroxy-succinimide (NHS). Afterward both lanes were saturated with 1M ethanolamide resulting in an actin-coated (lane 2) and a control lane (lane 1) for exclusion of non-specific binding. In general ligand partners were guided over both lanes. Sensograms of SYFO1 and AtFH8 were recorded in a running-buffer (10 mM HEPES pH 7.4, 150 mM NaCl, 3 mM EDTA, 10 mM MgCl2, 0.2 mM ADP, 0.5 mM DTT) at 25° with a flow rate of 30 μl/min. Starting with the lowest concentration, 5 different dilutions of a 1 μM protein solution (1:3 serial dilution) were injected sequentially for 120 s and dissociation was allowed for 600 s. The Chip surface was regenerated by an overnight wash with running buffer. Bound protein was determined as relative response units (RU) corrected for the unspecific binding to lane 1 (RULane2-RULane1). Kinetic constants were calculated by using the Biacore X100 Evaluation software.

#### Confocal Laser-Scanning Microscopy and FRAP

For imaging the NLS-GFP reporter module, sectioned nodules, protein localization and plasmolysis we used a Leica TCS SP8 confocal microscope equipped with a 20x HCX PL APO water immersion. GFP was excited with a White Light Laser (WLL) at 488 nm and the emission was detected at 500-550 nm. mCherry fluorescence was excited using a WLL at 561nm and emission was detected between 575-630 nm. Samples, co-expressing two fluorophores were imaged in sequential mode between frames.

FRAP analysis was conducted using a Zeiss LSM 880 Airyscan confocal microscope. For this, the VP (Virtual Pinhole) mode was adapted based on the fluorescence intensity of probe and Airyscan processing was performed using the ZEN (black edition) software package. The bleaching region, reference region and background region were selected at identical size. A pre-bleaching time of 5 s was chosen. Bleaching was set to stop upon the intensity dropping to 50% of the initial intensity before fluorescence recovery was recorded for 10 minutes. Using the FRAP data process package in ZEN (black version), the mobile fraction was calculated by the following equation (mobile fraction = *I*1/ *IE*), where *I*1 represents the dropped intensity and the *IE* represents the recovered intensity normalized to the reference intensity.

#### Root organ culture, protoplast extraction, and inoculation

Transgenic Root Organ Culture (ROC) of *M. truncatula* expressing SYFO1-GFP were obtained via hairy root transformation according to Boisson-Dernier et al.^31^ Fully transformed root segments were cut and initially transferred to M-Media plates^51^ containing Augmentin (Sigma). 1 g of amoxicillin–200 mg of clavulanic acid) (400 mg/l) for two weeks and then subcultured on plates supplemented with 200 mg/l Augmentin for additional two weeks to remove *A. rhizogenes* contamination. Plates were sealed with micropore tape and incubated at 24°C in dark. Afterward, the ROC was transferred to M-media plates without Augmentin to support faster tissue growth. Continuous expression of the transformation marker was monitored throughout the entire experiment.

Protoplasts were isolated from ROC, *syfo1-1* and *syfo1L-1* mutants by cutting the roots in small pieces of 2-5 mm length and processed as described previously.^52^ For inoculation, an *S. meliloti* culture was diluted in W5 solution^52^ to an OD_600_ of 0.05 before being added to the protoplasts. Protoplasts were additionally treated with isolated *S. meliloti* NFs at a concentration of 10 nM and the *S. meliloti* strains *exoH* and *exoY*^24^ and *nod A* ^25^) using the same ODs.

#### Actin phalloidin staining and plasma membrane FM4-64 staining

Phalloidin-based actin staining was performed according to a published protocol.^12^ In brief, *M. truncatula* roots were transferred into Fahraeus medium containing 300 μM MBS (m-maleimidobenzoyl- N-hydroxysuccinimide ester) for 30 minutes to stabilize the actin filaments. The material was then fixed in 2% formaldehyde in actin-stabilizing buffer (ASB) solution.^16^ Phalloidin was added to a final concentration of 16 μM and staining was performed in the dark for 30 minutes. Root-derived protoplasts were submerged in a FM4-64 solution with a final concentration of 20 μM and incubated on ice for 5-10 mins before imaging.

### Quantification and statistical analysis

All statistical tests have been carried out using the IBM SPSS Statistics Version 26 software. The corresponding tests and experimental details are mentioned in the corresponding figure legends. All graphical data plots have been generated using RStudio Version 1.3.1073 and fonts were adjusted using Adobe Illustrator CS7.

## Supplemental information

Document S1. Figures S1–S4 and Tables S1–S4

Document S2. Article plus supplemental information
