# Supplementary material for: Formin-mediated bridging of cell wall, plasma membrane, and cytoskeleton in symbiotic infections of Medicago truncatula
Source: Curr Biol. 2021 Jun 21;31(12):2712–2719.e5. doi: 10.1016/j.cub.2021.04.002 (PMC8231094; doi:10.1016/j.cub.2021.04.002)
Supplement: Document S1. Figures S1–S4 and Tables S1–S4 [file mmc1.pdf]

Current Biology, Volume *31*

Supplemental Information

Formin-mediated bridging of cell wall, plasma membrane, and cytoskeleton in symbiotic infections of *Medicago truncatula*

Pengbo Liang, Clara Schmitz, Beatrice Lace, Franck Anicet Ditengou, Chao Su, Eija Schulze, Julian Knerr, Robert Grosse, Jean Keller, Cyril Libourel, Pierre-Marc Delaux, and Thomas Ott

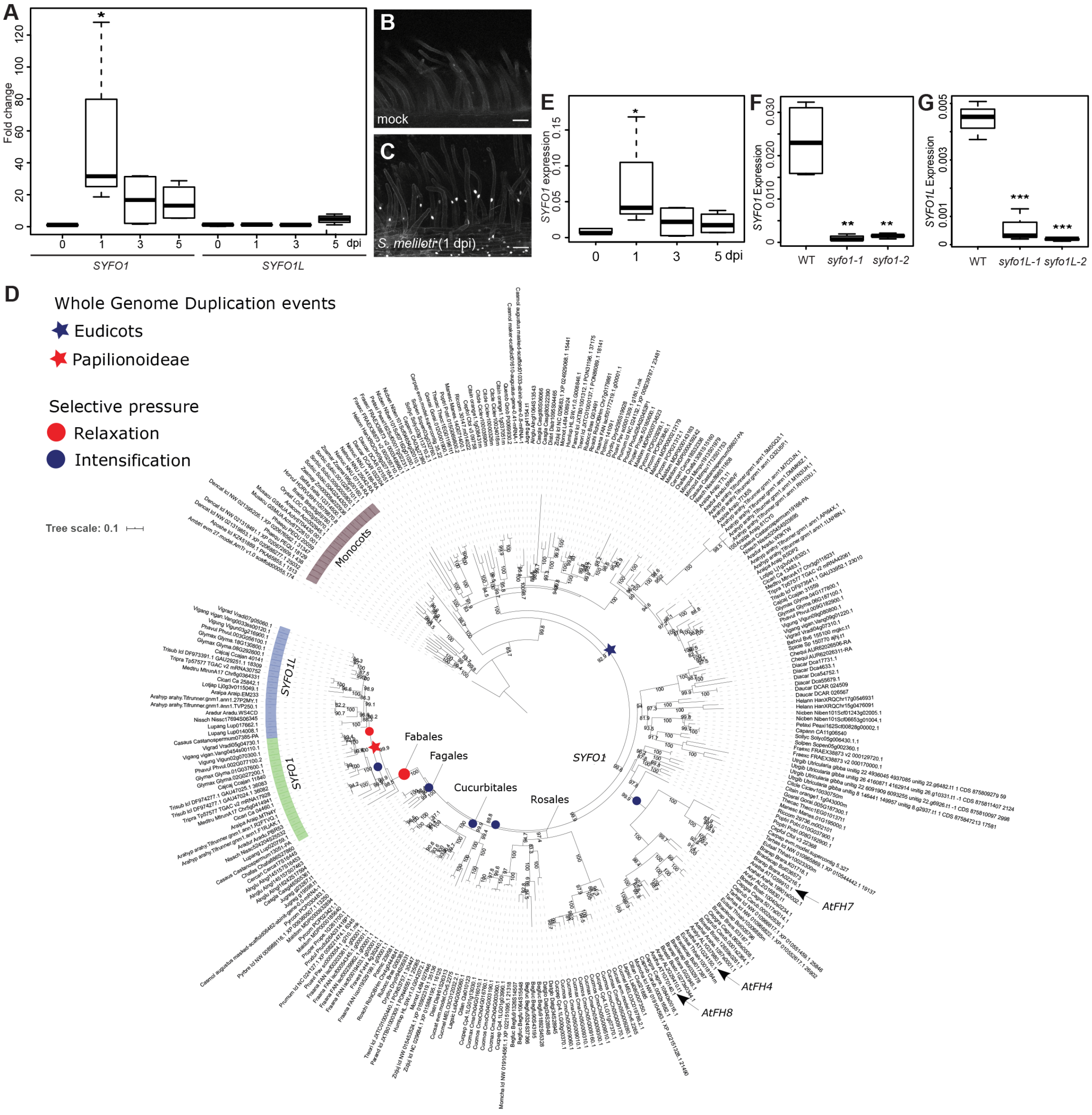

Figure S1. SYFO1 contributes to the symbiosis establishment. Related to Figure 1. (*A*) *SYFO1* and *SYFO1L* transcript levels before inoculation, at 1, 3 and 5 dpi. (*B-C*) Promoter activity was assessed with cellular resolution in transgenic *M. truncatula* root hairs by using the genetically encoded reporter *ProSYFO1:NLS-2xGFP*. (*D*) Maximum Likelihood tree (model: TIM3+F+R6; Log-likelihood: -124347.2322) of SYFO1 and SYFO1L family in 102 Angiosperm species. The tree was rooted on *Amborella trichopoda*. The Eudicots duplication leading to the emergence of the SYFO1/SYFO1L clade is indicated with a blue star and the *Papilionoideae* duplication from which SYFO1 and SYFO1L derived is indicated with a red star. Monocots, SYFO1 and SYFO1L clades are indicated by violet, green and blue ribbons respectively. Results of the selective pressure analysis are marked as follow: red dots indicate relaxation (K<1 and p-val < 0.01) of the selection in the clade while blue dots stand for an intensification of the selection (K>1 and p-val < 0.01). Details of selective pressure analysis are presented in Table S3. (*E*) Relative expression of *SYFO1* in inoculated roots. (*F-G*) Determining transcript levels in Tnt1 insertion lines. *SYFO1* transcript levels in wild-type R108, *syfo1-1*, *syfo1-2* at 1 dpi, and wild-type R108 *syfo1L-*

1, *syfo1L-2* without inoculation. Quantitative qRT-PCR was performed on cDNAs obtained from roots, with 4 biological replicates (each replicate contained roots from four independent plants). The graphs represent ΔΔCt values obtained by qRT-PCR relative to ubiquitin. Asterisks indicate a significant statistical difference based on a Tukey–Kramer multiple-comparison test with p-values of $p<0.05$ (*) $p<0.01$ (**) and $p<0.001$ (***). Scale bars indicate 50 µm (*B-C*).

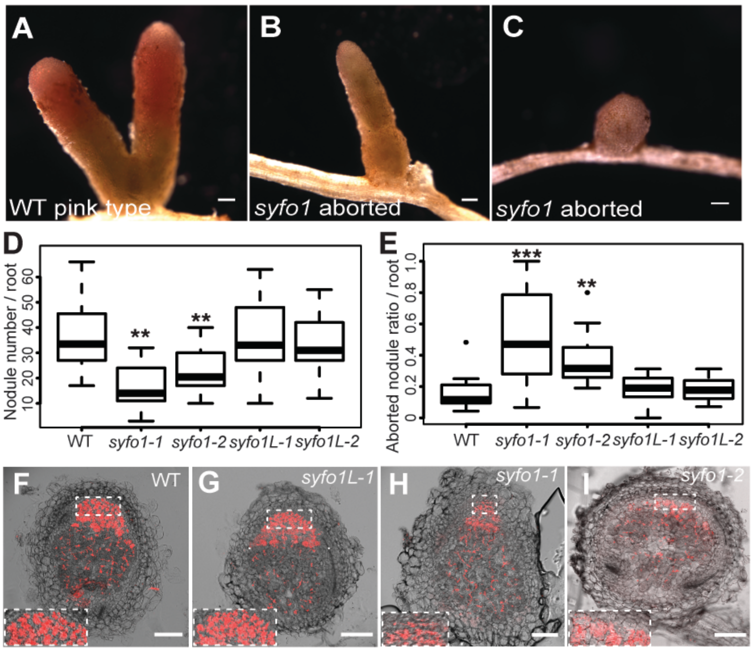

Figure S2. SYFO1 but not SYFO1L is required for nodulation. Related to Figure 1. (*A-C*) Nodule phenotypes observed on the different genotypes at 3 wpi with *S. meliloti* in open pots using WT R108 plants as a control. Quantification of nodule numbers (*D*) and the ratio of aborted/wild-type like nodules (*E*) (n=10 independent root systems per genotype). Semi-thin (60 µm) sections of agarose-embedded nodules representative for wild-type (*F*), *syfo1L* (*G*) and *syfo1* (*H-I*). The dashed rectangles indicate the magnified selected zones. Plants were grown in open pots and inoculated for 3 weeks with *S. meliloti* (red) prior to the analysis. Asterisks indicate a significant statistical difference based on a Tukey–Kramer multiple-comparison test with p-values <0.01 (**), < 0.001 (***). Data are shown as mean ± SE. Scale bars indicate 200 µm (*A-C*), 100 µm (*F-I*).

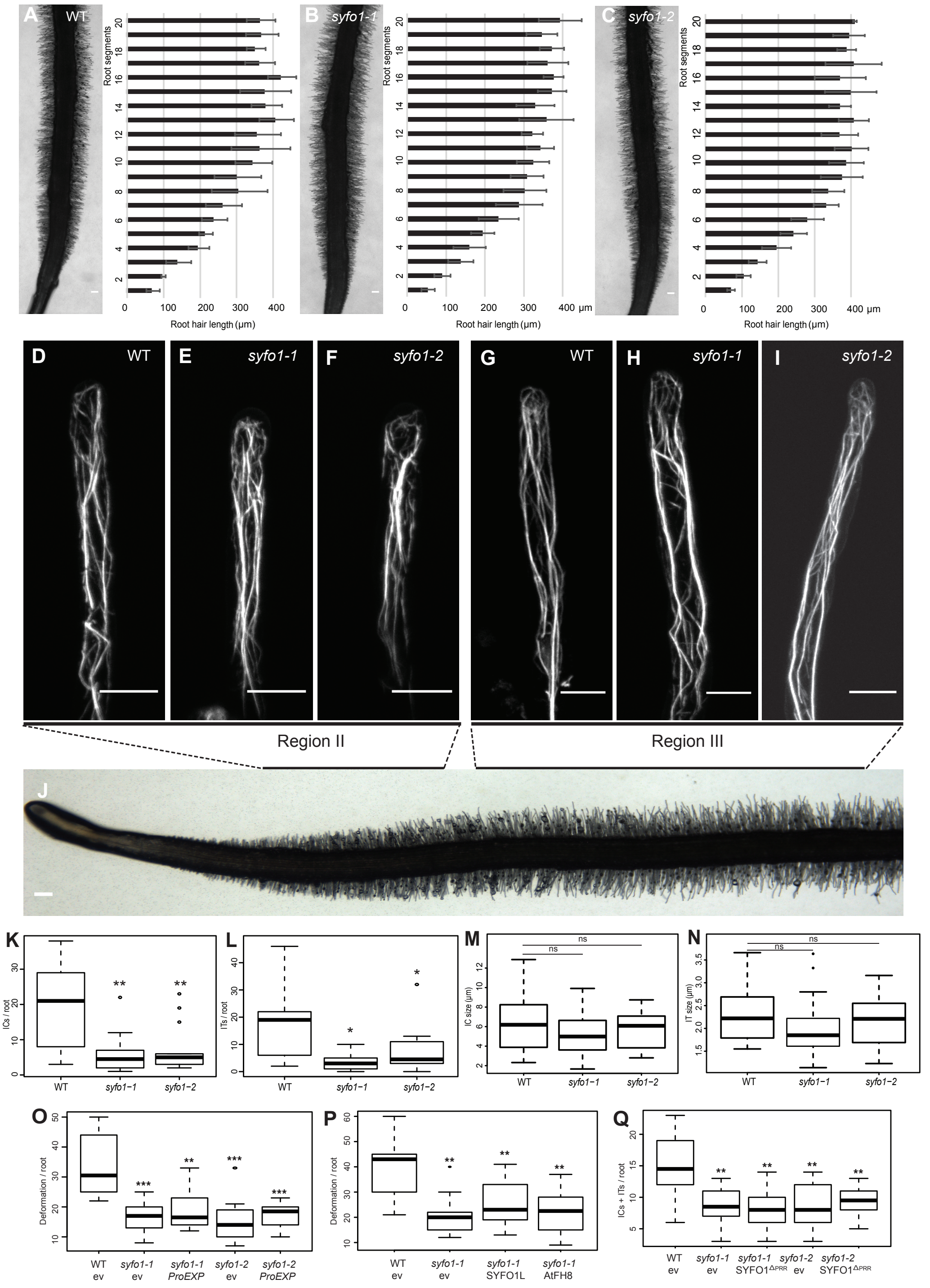

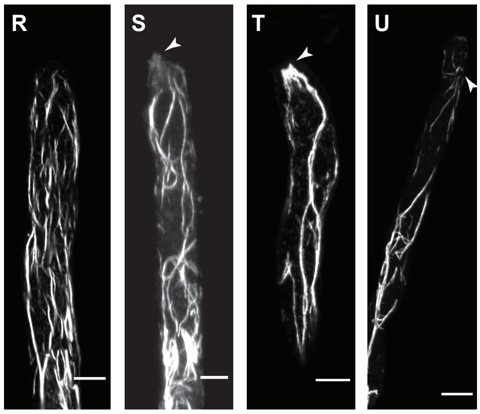

V

genotype	n_{polar}	n_{total}	% of n_{total}
WT	35	40	88
syfo1-1	11	35	31
syfo1-2	5	20	25

Figure S3. Analysis of actin and morphological patterns in symbiotically responding root hairs. Related to Figures 1 and 2.

Root hairs grow normally (*A-C*) and actin arrangements are indistinguishable in young, uninoculated root hairs of on *syfo1-1* and *syfo1-2* mutants (*D-J*). Plants were grown on vertical agar plates in the absence of rhizobia for 10 days prior to the analysis. Root hair length was measured in 20 consecutive root segments covering the designated infection zone (3 mm above the root hair tip). No statistically significant differences were found based on an ANOVA followed by a Fisher LSD test when comparing root hair length in between the corresponding root segment of the different genotypes. Data are shown as mean ± SE of 4 independent biological replicates with four roots being scored per experiment and genotype. Phalloidin staining of actin in root hairs of the differentiation zone (region II; *D-F*) and elongation zone (region III; *G-I*) as illustrated in *J*.

Dissecting early root hair responsiveness in *syfo1* mutants (*K-N*). The number of infection chambers (ICs) (*K*) and infection threads (ITs) (*L*) per root and the individual sizes of ICs (*M*) and ITs (*N*) were scored at 5 dpi in R108 wild-type as well as *syfo1-1* and *syfo1-2* mutant alleles. Asterisks indicate a significant statistical difference based on a Tukey–Kramer multiple-comparison test with p-values <0.05 (*) and 0.01 (**). Data are shown as mean ± SE and were obtained from 9-14 independent plants (*K, L*) and from 13 plant for (*M, N*). Complementation studies for *syfo1* mutants (*O-Q*). (*O*) *SYFO1* driven by the epidermis specific expansin promoter (*ProEXP*) failed to restore the deformation phenotype in *syfo1* mutants. (*P*) The *syfo1* root hair deformation phenotype could not be complemented by expressing *SYFO1L* and *AtFH8* driven by *SYFO1* promoter. (*Q*) The PRR segment in the SYFO1 extracellular domain is required for its function as a SYFO1$^{\Delta PRR}$ variant driven by the *SYFO1* promoter was not able to restore the infection thread and infection chamber phenotypes in *syfo1* mutants. ev= empty vector. Asterisks indicate a significant statistical difference based on a Tukey–Kramer multiple-comparison test with p-values < 0.01 (**) and 0.001 (***). Data were obtained from 10 independent roots for each analysis.

Analysis of actin polarization upon rhizobial inoculation (*R-V*). Actin patterns were visualized by Phalloidin staining of root hairs and observed under non-inoculated (*R*) and inoculated conditions after 2 dpi with *S. meliloti* (*S-U*). Representative images for longitudinal alignment of actin filaments in the absence of rhizobia in wild type (*R*), apically polarized actin (*S, T*) and polarized actin at the apical shank of root hairs (*U*) upon inoculation of wild-type root hairs. The appearance of polarized patterns (n_{polar}) as shown in panels *S-U* was scored on wild-type and *syfo1* mutant roots by absent/present calls of appearance on individual root systems (*V*). Arrowheads indicate sites of polarized actin accumulations. Scale bars indicate 200 µm (*A-C, G*). Scale bars indicate 10 µm (*D-I,* and *R-U*).

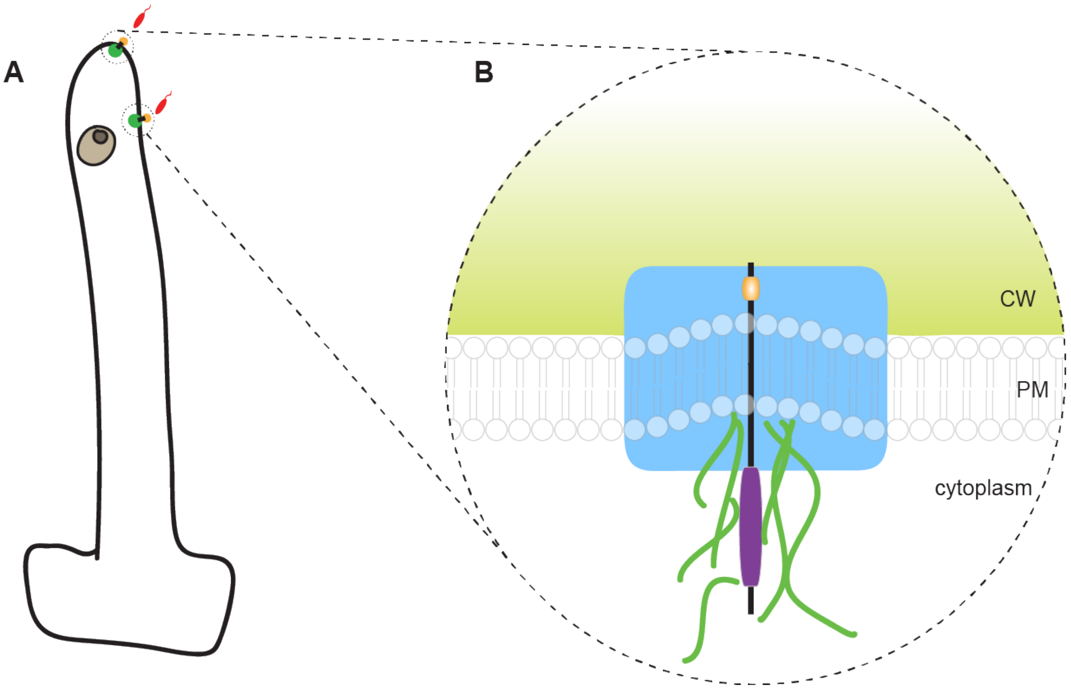

Figure S4. Proposed model for a SYFO1-mediated actin-plasma membrane-cell wall-continuum. Related to Figures 3 and 4. Our data suggest that SYFO1 establishes actin nucleation centres at the tip or the apical part of the shank of growing root hairs (green) upon inoculation with rhizobia (red) (*A*). (*B*) Association with the cell wall (CW; light green) by the proline-rich extracellular domain of SYFO1 (orange) laterally immobilizes SYFO1. Polar secretion of SYFO1 together with other membrane constituents and SYFO1-mediated actin nucleation (green filaments) may allow the formation of a polarity domain (bright blue). PM= plasma membrane. The FH1FH2 domain is depicted in purple.

Gene ID	Description
Medtr2g086990	formin-like 2 domain protein
Medtr4g045670	formin-like 2 domain protein
Medtr4g095780	formin-like 2 domain protein
Medtr8g027995	formin-like 2 domain protein
Medtr7g080920	formin-like 2 domain protein
Medtr4g087890	formin-like 2 domain protein
Medtr5g015690	formin-like 2 domain protein
Medtr5g036540	formin-like 2 domain protein
Medtr3g078623	formin-like 2 domain protein
Medtr1g083260	formin-like 2 domain protein
Medtr2g082190	formin-like 2 domain protein
Medtr4g081410	formin-like 2 domain protein
Medtr0094s0010	formin-like 2 domain protein
Medtr8g062830	formin-like 2 domain protein
Medtr3g037080	actin-binding FH2 (formin-like) protein, putative
Medtr4g109040	actin-binding FH2 (formin-like) protein
Medtr1g013800	actin-binding FH2 (formin 2) family protein
Medtr4g131020	actin-binding FH2 (formin 2) family protein
Medtr4g109040	actin-binding FH2 (formin-like) protein
Medtr5g026645	actin-binding FH2 (formin-like) protein

Table S1: FH2-domain containing proteins identified in the *Medicago truncatula* genome. Related to Figure 1.

Abbreviation	Species	Order	Family	Reference	N-fixing
Alnglu	*Alnus glutinosa*	Fagales	Betulaceae	10.1126/science.aat1743	1
Ambtri	*Amborella trichopoda*	Amborellales	Amborellaceae	10.1126/science.1241089	0
Anacom	*Ananas comosus*	Bromeliales	Bromeliaceae	10.1038/ng.3435	0
Aposhe	*Apostasia shenzhenica*	Asparagales	Orchidaceae	10.1038/nature23897	0
Arahal	*Arabidopsis halleri*	Brassicales	Brassicaceae	Phytozome	0
Aralyr	*Arabidopsis lyrata*	Brassicales	Brassicaceae	10.1038/ng.807	0
Aratha	*Arabidopsis thaliana*	Brassicales	Brassicaceae	10.1093/nar/gkr1090	0
Aradur	*Arachis duranensis*	Fabales	Fabaceae	10.1038/ng.3517	1
Arahyp	*Arachis hypogaea*	Fabales	Fabaceae	https://peanutbase.org/data/public/Arachis_hypogaea/Tifrunner.gnm1.KYV3/	1
Araipa	*Arachis ipaensis*	Fabales	Fabaceae	10.1038/ng.3517	1
Begfuc	*Begonia fuchsioides*	Cucurbitales	Begoniaceae	10.1126/science.aat1743	0
Betvul	*Beta vulgaris ssp. vulgaris KWS2320*	Caryophyllales	Amaranthaceae	10.1038/nature12817	0
Boestr	*Boechera stricta*	Brassicales	Brassicaceae	Phytozome	0
Bradis	*Brachypodium distachyon*	Poales	Poaceae	10.1038/nature08747	0
Braolecap	*Brassica oleraceae capitata*	Brassicales	Brassicaceae	10.1038/ncomms4930	0
Brarap	*Brassica rapa FPsc*	Brassicales	Brassicaceae	Phytozome	0
Cajcaj	*Cajanus cajan*	Fabales	Fabaceae	10.1038/nbt.2022.	1
Capgra	*Capsella grandiflora*	Brassicales	Brassicaceae	10.1038/ng.2669	0
Caprub	*Capsella rubella*	Brassicales	Brassicaceae	10.1038/ng.2669	0
Capann	*Capsicum annuum cvCM334*	Solanales	Solanaceae	10.1038/ng.2877	0
Carpap	*Carica papaya*	Brassicales	Caricaceae	10.1038/nature06856	0
Casmol	*Castanea mollissima*	Fagales	Fagaceae	https://www.hardwoodgenomics.org/organism/Castanea/mollissima	0
Casaus	*Castanospermum australe*	Fabales	Fabaceae	10.1126/science.aat1743	0
Casgla	*Casuarina glauca*	Fagales	Casuarinaceae	10.1126/science.aat1743	1
Cepfol	*Cephalotus follicularis*	Oxalidales	Cephalotaceae	10.1038/s41559-016-0059	0
Cercan	*Cercis canadensis*	Fabales	Caesalpiniaceae	10.1126/science.aat1743	0
Chafas	*Chamaecrista fasciculata*	Fabales	Fabaceae	10.1126/science.aat1743	1
Chequi	*Chenopodium quinoa*	Caryophyllales	Amaranthaceae	10.1038/nature21370	0
Cicari	*Cicer arietinum ICC4958*	Fabales	Fabaceae	10.1038/srep12806	1
Citlan	*Citrullus lanatus subsp. vulgaris 97103*	Cucurbitales	Cucurbitaceae	10.1038/ng.2470	0
Citcle	*Citrus clementina*	Sapindales	Rutaceae	10.1038/nbt.2906	0
Citsin	*Citrus sinensis*	Sapindales	Rutaceae	10.1038/nbt.2906	0
Cucmel	*Cucumis melo*	Cucurbitales	Cucurbitaceae	10.1073/pnas.1205415109	0
Cucsat	*Cucumis sativus PI183967*	Cucurbitales	Cucurbitaceae	10.1038/ng.2801	0
Cucmax	*Cucurbita maxima*	Cucurbitales	Cucurbitaceae	10.1016/j.molp.2017.09.003	0
Cucmos	*Cucurbita moschata*	Cucurbitales	Cucurbitaceae	10.1016/j.molp.2017.09.004	0
Cucpep	*Cucurbita pepo*	Cucurbitales	Cucurbitaceae	http://cucurbitgenomics.org/organism/13	0
Datglo	*Datisca glomerata*	Cucurbitales	Dasticaceae	10.1126/science.aat1743	0
Daucar	*Daucus carota*	Apiales	Apiaceae	10.1038/ng.3565	0
Dencat	*Dendrobium catenatum*	Asparagales	Orchidaceae	10.1038/nature23897	0
Diacar	*Dianthus caryophyllus*	Caryophyllales	Caryophyllaceae	10.1093/dnares/dst053	0
Distri	*Discaria trinervis*	Rosales	Rhamnaceae	10.1126/science.aat1743	1
Drydru	*Dryas drummondii*	Rosales	Rosaceae	10.1126/science.aat1743	1
Eutsal	*Eutrema salsugineum*	Brassicales	Brassicaceae	10.3389/fpls.2013.00046	0
Fraves	*Fragaria vesca*	Rosales	Rosaceae	10.1038/ng.740	0
Fraana	*Fragaria x ananassa*	Rosales	Rosaceae	https://www.rosaceae.org/species/fragaria/fragaria_x_ananassa	0
Fraexc	*Fraxinus excelsior*	Lamiales	Oleaceae	10.1038/nature20786	0

Glymax	*Glycine max*	Fabales	Fabaceae	10.1038/nature08670	1
Gosrai	*Gossypium raimondii*	Malvales	Malvaceae	10.1038/nature11798	0
Helann	*Helianthus annuus*	Asterales	Asteraceae	10.1038/nature22380	0
Horvul	*Hordeum vulgare*	Poales	Poaceae	10.1038/nature22043 / 10.1038/sdata.2017.44	0
Humlup	*Humulus lupulus*	Rosales	Cannabaceae	10.1093/pcp/pcu169	0
Jugreg	*Juglans regia*	Fagales	Juglandaceae	10.1111/tpj.13207	0
Lagsic	*Lagenaria siceraria*	Cucurbitales	Cucurbitaceae	10.1111/tpj.13722	0
Lotjap	*Lotus japonicus*	Fabales	Fabaceae	10.1093/dnares/dsn008	1
Lupang	*Lupinus angustifolius*	Fabales	Fabaceae	10.1111/pbi.12615	1
Maldom	*Malus domestica*	Rosales	Rosaceae	https://www.rosaceae.org/species/malus/malus_x_domestica/genome_v3.0.a0	0
Manesc	*Manihot esculenta*	Malpighiales	Euphorbiaceae	10.1038/nbt.3535	0
Medtru	*Medicago truncatula*	Fabales	Fabaceae	10.1038/s41477-018-0286-7	1
Mimpud	*Mimosa pudica*	Fabales	Fabaceae	10.1126/science.aat1743	1
Momcha	*Momordica charantia*	Cucurbitales	Cucurbitaceae	https://www.ncbi.nlm.nih.gov/assembly/GCF_001995035.0	0
Mornot	*Morus notabilis*	Rosales	Moraceae	10.1038/ncomms3445	0
Musacu	*Musa acuminata*	Zingiberales	Zingiberaceae	10.1093/database/bat035	0
Nelnuc	*Nelumbo nucifera*	Proteales	Nelumbonaceae	10.1186/gb-2013-14-5-r41	0
Nicben	*Nicotiana benthamiana*	Solanales	Solanaceae	10.1094/MPMI-06-12-0148-TA	0
Nissch	*Nissolia schottii*	Fabales	Fabaceae	10.1126/science.aat1743	0
Orysat	*Oryza sativa*	Poales	Poaceae	10.1093/nar/gkl976	0
Parand	*Parasponia andersonii*	Rosales	Cannabaceae	10.1073/pnas.1721395115	1
Petaxi	*Petunia axillaris*	Solanales	Solanaceae	10.1038/nplants.2016.74	0
Phaequ	*Phalaenopsis equestris*	Asparagales	Orchidaceae	10.1038/nature23897	0
Phavul	*Phaseolus vulgaris*	Fabales	Fabaceae	10.1038/ng.3008	1
Poptri	*Populus trichocarpa*	Malpighiales	Salicaceae	10.1126/science.1128691	0
Potmic	*Potentilla micrantha*	Rosales	Rosaceae	10.1093/gigascience/giy010	0
Pruavi	*Prunus avium*	Rosales	Rosaceae	10.1093/dnares/dsx020	0
Prudul	*Prunus dulcis*	Rosales	Rosaceae	https://www.rosaceae.org/analysis/294	0
Prumum	*Prunus mume*	Rosales	Rosaceae	https://www.ncbi.nlm.nih.gov/assembly/GCF_000346735.0	0
Pruper	*Prunus persica*	Rosales	Rosaceae	10.1038/ng.2586	0
Pyrcom	*Pyrus communis*	Rosales	Rosaceae	10.1371/journal.pone.0092644	0
Pyrbre	*Pyrus x bretschneideri*	Rosales	Rosaceae	https://www.ncbi.nlm.nih.gov/assembly/GCF_000315295.0	0
Querob	*Quercus robur*	Fagales	Fagaceae	10.1111/1755-0998.12425	0
Riccom	*Ricinus communis*	Malpighiales	Euphorbiaceae	10.1038/nbt.1674	0
Roschi	*Rosa chinensis*	Rosales	Rosaceae	10.1038/s41588-018-0110-3	0
Rubocc	*Rubus occidentalis*	Rosales	Rosaceae	10.1111/tpj.13215	0
Setita	*Setaria italica*	Poales	Poaceae	10.1038/nbt.2196	0
Sollyc	*Solanum lycopersicum*	Solanales	Solanaceae	10.1038/nature11119	0
Solpen	*Solanum pennellii*	Solanales	Solanaceae	10.1038/ng.3046	0
Sorbic	*Sorghum bicolor*	Poales	Poaceae	10.1111/tpj.13781	0
Spiole	*Spinacia oleracea*	Caryophyllales	Amaranthaceae	http://bvseq.molgen.mpg.de/Genome/Download/Spinach/	0
Spipol	*Spirodela polyrhiza*	Alismatales	Araceae	10.1038/ncomms4311	0
Tarhas	*Tarenaya hassleriana*	Brassicales	Cleomaceae	10.1105/tpc.113.113480	0
Thecac	*Theobroma cacao*	Malvales	Malvaceae	10.1186/gb-2013-14-6-r53	0
Treori	*Trema orientalis*	Rosales	Cannabaceae	10.1073/pnas.1721395115	0
Tripra	*Trifolium pratense*	Fabales	Fabaceae	10.1038/srep17394	1
Trisub	*Trifolium subterraneum*	Fabales	Fabaceae	https://www.ncbi.nlm.nih.gov/assembly/GCA_001742945.1#/st	1
Utrgib	*Utricularia gibba*	Lamiales	Lentibulariaceae	10.1073/pnas.1702072114	0

Vigang	*Vigna angularis*	Fabales	Fabaceae	10.1038/srep080669	1
Vigrad	*Vigna radiata*	Fabales	Fabaceae	10.1038/ncomms6443	1
Vigung	*Vigna unguiculata*	Fabales	Fabaceae	Phytozome	1
Zeamay	*Zea mays PH207*	Poales	Poaceae	10.1105/tpc.16.00353	0
Zizjuj	*Ziziphus jujuba cv. Dongzao*	Rosales	Rhamnaceae	10.1038/ncomms6315	0
Zosmar	*Zostera marina*	Alismatales	Zosteraceae	10.1038/nature16548	0

Table S2: List of species investigated in this study. Related to STAR Methods. The N-fixing column indicates if species can (1) or not (0) establish root nodule nitrogen-fixing symbiosis.

Tree	Analyzed sequences	Analyzed sites	Tested branches	Branch model - RELAX			
				K	LRT	*p-val*	Selective pressure
Syfo1/1L/3 angiosperm tree	280	766	*Brassicaceae*	**9,34**	**202,89**	**0,00E+00**	intensification
			Cucurbitales	**8,71**	**47,34**	**5,97E-12**	intensification
			Fabales	**0,54**	**22,32**	**2,31E-06**	relaxation
			Fagales	**1,73**	**27,97**	**1,23E-07**	intensification
			Rosales	0,96	4,40	3,59E-02	
			NFN clade *(Cuc, Fab, Fag and Ros)*	**1,09**	**15,82**	**6,97E-05**	intensification
			Syfo1 clade	**6,46**	**52,89**	**3,53E-13**	intensification
			Syfo1 clade infection threads forming species	0,84	4,34	3,70E-02	
			Syfo1L clade	**0,45**	**40,61**	**1,86E-10**	relaxation
			Syfo1L clade infection threads forming species	0,96	0,840	3,60E-01	
Syfo1/1L NFN tree	107	779	Infection threads forming species	**0,25**	**18,63**	**1,59E-05**	relaxation
			Syfo1 Infection threads forming species	0,87	5,97	1,50E-02	
			Syfo1L Infection threads forming species	0,91	5,96	1,50E-02	
Syfo1/1L *Fabales* tree	41	758	Infection threads forming species	**1,43**	**16,76**	**4,24E-05**	intensification
Syfo1 *Fabales* tree	19	800	No Infection threads forming species	0,95	0,62	4,30E-01	
Syfo1L *Fabales* tree	20	772	No Infection threads forming species	**0,66**	**7,29**	**7,00E-03**	relaxation

Table S3: Results of the branch model analysis for signature of selection acting in the SYFO1/SYFO1L angiosperm clade on different branches of interest. Related to STAR Methods. The column tree indicates which species and corresponding phylogenetic tree was used to run the branch model RELAX. Analyzed sequences and sites indicated the number of sequences and sites analyzed corresponding to the species/tree selected. The tested branches column indicates which clade was targeted as 'Foreground' to compare selective pressure against the rest of the tree. K represent the selection intensity parameter where a significant result of k>1 indicates that selection strength has been intensified along the test branches, and a significant result of k<1 indicates that selection strength has been relaxed along the test branches. LRT represent the Likelihood Ratio Test to comparing the alternative and null models from RELAX with the corresponding p-value. Bold p-values indicate significant at 0.01 threshold.

Primer	Sequence (5'-3')	Used for	References or referenced ID
gt-SYFO1-F	AGCCACAATAACACAACCTCCT	Genotyping	Medtr5g036540.1
gt-SYFO1-R	GCCTTTGTTGTCTTCTTTTTCTGC	Genotyping	Medtr5g036540.1
gt-SYFO2-F	GCCAAAACAATTCTCCACAAAAC	Genotyping	Medtr8g062830.1
gt-SYFO2-R	TCCCAAGAAATCCTTAACTATACCA	Genotyping	Medtr8g062830.1
TNT1-F	GTAGAGAATAGGTAAGGTGCT	Genotyping	[S1]
TNT1-R	TGTAGCACCGAGATACGGTAATTAACAAGA	Genotyping	[S1]
TNT1-R1	TGTAGCACCGAGATACGGTAATTAACAAGA	Genotyping	[S1]
qUbi-F	TTGTGTGTTGAATCCTAAGCA	Housekeeping gene for qRT-PCR	[S2]
qUbi-R	CAAGACCCATGCAACAAGTTC	Housekeeping gene for qRT-PCR	[S2]
qSYFO1-F	AGTAGCAGCAGCAGCGGAGA	qRT-PCR for candidate prescreen and mutant transcript check	Medtr5g036540.1
qSYFO1-R	TCCTTGCTGAGAGTGCTGAGA	qRT-PCR for candidate prescreen and mutant transcript check	Medtr5g036540.1
qSYFO2-F	GTTCAGAAACAAGGCTTTCAATCTCA	qRT-PCR for candidate prescreen	Medtr8g062830.1
qSYFO2-R	GATCATTGTTGGATTCTTGCATCATG	qRT-PCR for candidate prescreen	Medtr8g062830.1
qSYFO2-F2	GGTGAGAGAGGAAATTTTGTG	qRT-PCR for mutant transcript check	Medtr8g062830.1
qSYFO2-R2	ACCCCCACATTCATAATACTG	qRT-PCR for mutant transcript check	Medtr8g062830.1
pSYFO1-F	ATGGTCTCAGCGGAGGGGCATGTTAATCACC	Cloning	Medtr5g036540.1
pSYFO1-R	TAGGTCTCTCAGAGAATGAAGAGGAACAAAGAT	Cloning	Medtr5g036540.1
gSYFO1-F	ATGGTCTCACACCATGTCTTTGAACATGATGTTTC	Cloning	Medtr5g036540.1
gSYFO1-R	TAGGTCTCTCCTTAAACTTTGTCTTAGGTGTTTTC	Cloning	Medtr5g036540.1

SYFO1^{ECD}-F	ATGGTCTCACACCATGTCTTTGAACATGATGTTTC	Cloning	Medtr5g036540.1
SYFO1^{ECD}-R	TAGGTCTCTCCTTTCACTTACTATTTGTTGAAGAATTTGT	Cloning	Medtr5g036540.1

Table S4: List of primers used in this study. Related to STAR Methods. Abbreviations: Forward (F) and reverse (R) primers, Ubiquitin (Ubi)

SUPPLEMENTAL REFERENCES

[S1] Tadege, M., Wen, J.Q., He, J., Tu, H.D., Kwak, Y., Eschstruth, A., Cayrel, A., Endre, G., Zhao, P.X., Chabaud, M., et al. (2008). Large-scale insertional mutagenesis using the Tnt1 retrotransposon in the model legume *Medicago truncatula*. Plant J. *54*, 335-347.

[S2] Satge, C., Moreau, S., Sallet, E., Lefort, G., Auriac, M.C., Rembliere, C., Cottret, L., Gallardo, K., Noirot, C., Jardinaud, M.F., et al. (2016). Reprogramming of DNA methylation is critical for nodule development in *Medicago truncatula*. Nat. Plants *2*, 1-10.
